# Cheonggukjang-Derived *Bacillus* Strains Exhibit Protective Effects Against HCl/Ethanol-Induced Gastric Injury Associated with Reduced Inflammatory Responses

**DOI:** 10.3390/metabo16070481

**Published:** 2026-07-08

**Authors:** Yun-Seong Lee, Sooah Kim

**Affiliations:** 1 Nain Healthcare Co., Ltd., 3, Seodong-ro 20-gil, Iksan 54613, Republic of Korea; iyoonseong@daum.net; 2 Department of Food and Nutrition, Jeonju University, Jeonju 55069, Republic of Korea

**Keywords:** gastroprotective effect, gastric mucosal injury, *Bacillus* strains, anti-inflammatory, NF-κB signaling

## Abstract

Background/Objectives: Hydrochloric acid (HCl)/ethanol-induced gastric mucosal injury is closely associated with oxidative stress and inflammatory responses. Probiotics are promising therapeutic agents for gastrointestinal disorders. Therefore, this study evaluated the preventive effects of Cheonggukjang-derived *Bacillus* strains on HCl/ethanol-induced gastric mucosal injury in a rat model (*n* = 5 per group), with emphasis on inflammatory mechanisms. Methods: Eight-week-old male Sprague–Dawley rats were orally administered *Bacillus amyloliquefaciens* C393 1.0 × 10^9^ CFU (B393), *Bacillus subtilis* C439 1.0 × 10^9^ CFU (B439), or *B. subtilis* C512 1.0 × 10^9^ CFU (C512). Gastric injury was induced by oral administration of HCl/ethanol (150 mM HCl in 60% ethanol). Results: HCl/ethanol exposure significantly increased symptom scores, gastric mucosal lesion area, and histopathological damage. Pretreatment with B393 and B439 significantly attenuated these alterations. Moreover, HCl/ethanol administration increased gastric secretion volume and decreased gastric pH, both of which were significantly normalized by B393 and B439. Serum levels of pro-inflammatory cytokines, including tumor necrosis factor-α and interleukin-6, were significantly elevated following HCl/ethanol exposure but were markedly reduced by B393 and B439. Immunohistochemical analysis demonstrated that nuclear factor-kappa B activation in gastric tissues was significantly suppressed in these groups. Conclusions: These findings indicate that Cheonggukjang-derived *Bacillus* strains exert strain-specific gastroprotective effects possibly by suppressing NF-κB-mediated inflammatory signaling.

## 1. Introduction

Gastric mucosal injury is a common gastrointestinal disorder that occurs when aggressive factors, such as gastric acid, ethanol, nonsteroidal anti-inflammatory drugs, and *Helicobacter pylori*, overwhelm the intrinsic defense mechanisms of the gastric mucosa [1]. The gastric mucosa is normally protected by coordinated defense systems, including the mucus–bicarbonate barrier, prostaglandin-mediated cytoprotection, epithelial restitution, and adequate mucosal blood flow, which maintain mucosal integrity under physiological conditions [2]. Among various experimental models, hydrochloric acid (HCl)/ethanol-induced gastric injury is widely used because it effectively mimics acute gastric mucosal damage characterized by epithelial disruption, hemorrhage, and inflammatory infiltration [3]. This model reflects a pathological condition in which chemical injury disrupts mucosal defense systems and induces rapid tissue damage, making it suitable for evaluating gastroprotective agents [4].

Oxidative stress plays a central role in the pathogenesis of HCl/ethanol-induced gastric injury, as excessive generation of reactive oxygen species (ROS) leads to lipid peroxidation, cellular membrane damage, and apoptosis in gastric tissues [5]. Ethanol rapidly penetrates the gastric mucosa and induces microvascular disturbances and ROS overproduction, which further aggravates mucosal damage in the presence of gastric acid [6]. These oxidative events activate inflammatory signaling pathways, particularly the nuclear factor-kappa B (NF-κB) pathway, a key regulator of inflammation [7]. NF-κB signaling controls the transcription of various pro-inflammatory mediators and is critically involved in the progression of gastric mucosal injury [8]. Activation of NF-κB induces increased production of pro-inflammatory cytokines, including tumor necrosis factor-α (TNF-α) and interleukin-6 (IL-6), thereby amplifying inflammatory responses and exacerbating tissue damage [9]. Cytokines play central roles in mediating inflammatory processes and are strongly associated with the severity of gastric injury [10]. Oxidative stress and inflammation are closely interconnected processes, where ROS can activate NF-κB signaling, creating a vicious cycle that perpetuates gastric mucosal damage [11]. Further, ROS act as key signaling molecules in inflammation and contribute to immune response regulation [12].

In recent years, probiotics have emerged as promising therapeutic agents for gastrointestinal disorders due to their ability to modulate host immunity and regulate inflammatory signaling pathways [13]. The beneficial effects of probiotics are supported by international consensus, highlighting their roles in maintaining gut homeostasis and immune regulation [14]. Probiotic bacteria interact with host cells and influence immune responses through multiple mechanisms, including modulation of cytokine production and epithelial barrier function [15]. Furthermore, probiotics can influence gut microbiota composition and contribute to the regulation of inflammatory diseases [16]. Among various probiotic candidates, *Bacillus* species have attracted increasing attention due to their spore-forming ability, which confers high stability and resistance to the harsh gastrointestinal environment [17]. *Bacillus* probiotics improve intestinal barrier function and modulate immune responses, contributing to their therapeutic potential [18]. Specifically, *Bacillus subtilis* strains can regulate immune responses and exert anti-inflammatory effects by modulating signaling pathways [19]. Moreover, *Bacillus*-derived exopolysaccharides reportedly induce anti-inflammatory macrophage polarization and suppress inflammatory responses [20]. In addition, *B. subtilis*-derived bioactive components have been reported to induce anti-inflammatory macrophage responses and reduce inflammatory mediator production, supporting the immunomodulatory potential of *Bacillus* strains [21]. Further, *Bacillus*-derived metabolites can reportedly modulate the MAPK and NF-κB pathways, thereby contributing to anti-inflammatory effects [22]. Additionally, probiotic *Bacillus* strains have been reported to alleviate intestinal inflammation and improve barrier function in experimental models [23]. Even inactivated *Bacillus* preparations have shown immunomodulatory effects, suggesting that bacterial components can regulate host inflammatory responses [24].

Cheonggukjang, a traditional Korean fermented soybean product, is a rich source of diverse *Bacillus* species, including *B. subtilis* and *Bacillus amyloliquefaciens* [25]. The physiological functions of Cheonggukjang are influenced by strain-specific characteristics of the resident *Bacillus* species, which determine their bioactive properties [26]. Based on these findings, it is hypothesized that Cheonggukjang-derived *Bacillus* strains may exert gastroprotective effects against HCl/ethanol-induced gastric injury through modulation of oxidative stress and suppression of NF-κB-mediated inflammatory responses.

To test this, the present study aimed to investigate the protective effects of *B. amyloliquefaciens* C393, *B. subtilis* C439, and *B. subtilis* C512 on HCl/ethanol-induced gastric mucosal injury in rats, with a particular focus on inflammatory cytokines and NF-κB signaling.

## 2. Materials and Methods

### 2.1. Experimental Animals and Group Composition

The experimental animals used herein were 7-week-old male Sprague–Dawley (SD) rats purchased from Samtaco Co., Ltd. (Osan, Republic of Korea) and used after a one-week acclimatization period. Animals were randomly assigned to each experimental group following the acclimatization period. Investigators involved in histopathological evaluation and image analysis were blinded to treatment allocation during data collection and analysis to minimize experimental bias. Only male Sprague–Dawley rats were used in this study to minimize hormonal variability associated with the estrous cycle, which may influence inflammatory and oxidative stress responses. In addition, the use of male rats is consistent with previous HCl/ethanol-induced gastric injury models, allowing improved experimental consistency and comparison with earlier studies. The average weight of the rats was 250–300 g, and they were fasted for approximately 24 h, from 9:00 a.m. the day before the experiment until 9:00 a.m. on the day of the experiment. The light and dark cycles in the animal housing room were adjusted at 12 h intervals, and the temperature was maintained at 23 ± 2 °C and humidity at 50–60%. Sample size was determined based on previous studies using HCl/ethanol-induced gastric injury models that demonstrated statistically significant differences with comparable group sizes. In addition, animal numbers were minimized in accordance with ethical guidelines for animal experimentation and the ARRIVE recommendations. This animal experiment was conducted in compliance with the Wonkwang University Guide for Animal Experimentation and approved by the Wonkwang University Animal Ethics Committee (Approval NO. WKU25-87).

### 2.2. Preparation of an Acute Gastritis Model

The strains used in this study were isolated from traditional Cheonggukjang and identified by 16S rRNA gene sequencing. Based on sequence analysis, the isolates were identified as *Bacillus amyloliquefaciens* C393, *Bacillus subtilis* C439, and *B. subtilis* C512. After fasting for 24 h, the animals were orally administered *Bacillus* strains (1.0 × 10^9^ CFU) or distilled water according to the assigned experimental groups. One hour after pretreatment, acute gastric mucosal injury was induced by oral administration of 1 mL HCl/ethanol solution (150 mM HCl in 60% ethanol) according to the method of Mizui and Doteuchi [27]. Clinical symptoms were evaluated 30 min after HCl/ethanol administration to assess the preventive effects of the *Bacillus* strains against acute gastric injury. The animal grouping for treatment is shown in Table 1. The present study evaluated only a single dose (1.0 × 10^9^ CFU) of *Bacillus* strains based on previous probiotic studies demonstrating biological efficacy in rodent models. However, the absence of a dose–response analysis limits determination of the minimum effective dose and potential dose-dependent effects. Further studies evaluating multiple dose ranges are warranted.

### 2.3. Clinical Symptom Evaluation

Movement and symptoms were observed 30 min after administering HCl/ethanol (150 mM HCl + 60% ethanol). Based on clinical evaluation criteria, such as activity level and respiration, the results were calculated by averaging three measurements taken by three observers under the same conditions for 1 h. Scores were assigned based on general clinical signs, including locomotor activity, response to stimulation, grooming behavior, and respiratory changes, as previously recommended for the recognition and assessment of pain, distress, and discomfort in experimental animals [28].

### 2.4. Inhibition Rate of Acute Gastritis and Gastric Damage

One hour after *Bacillus* strain administration, 1 mL of HCl/ethanol (150 mM HCl + 60% ethanol) was orally administered to each experimental animal, and the animals were euthanized with ether 1 h later. Specifically, the stomach was dissected along the greater curvature, the inside of the stomach was flushed with saline solution, and the greater curvature was photographed and analyzed using an image analysis software (NIH, Bethesda, MD, USA) to measure the area of inflammation and ulceration. Gastric damage inhibition was expressed as the inhibition ratio [inhibition ratio (%)] determined according to a previous study using the following formula [29]:

$$Inhibition\;Ratio\;(\%)=\frac{Damaged\;Area\;of\;Control\;-Damaged\;Area\;of\;Sample}{Damaged\;Area\;of\;Control}\times 100$$

### 2.5. Measurement of Gastric pH and Gastric Juice Volume

The effects of Cheonggukjang-derived *Bacillus* strains on gastric secretion were evaluated by measuring gastric pH and gastric juice volume [30]. Following the induction of gastric mucosal injury with HCl/ethanol, the animals were sacrificed under anesthesia, and the stomachs were immediately excised. Gastric contents were carefully collected and centrifuged at 3000× *g* for 10 min to remove debris. The volume of gastric juice was measured using a graduated tube and expressed as mL per rat. The pH of the gastric juice was determined using a calibrated digital pH meter (Orion Star A211, Thermo Fisher Scientific, Waltham, MA, USA). All measurements were performed immediately after sample collection to minimize pH variation.

### 2.6. Cytokine Levels

Blood samples were collected at animal sacrifice, allowed to clot at room temperature, and centrifuged to obtain serum. Serum levels of TNF-α and interleukin (IL)-6 were measured using commercially available ELISA kits (TNF-α: Cat. No. RTA00; IL-6: Cat. No. R6000B, R&D Systems, Minneapolis, MN, USA) according to the manufacturers’ instructions. Briefly, standards and serum samples were added to antibody-coated wells and incubated for the recommended period, followed by sequential washing, incubation with detection conjugate, substrate development, and reaction termination. Absorbance was measured at 450 nm using a microplate reader (SpectraMax M2, Molecular Devices, San Jose, CA, USA). Cytokine concentrations were calculated from standard curves generated for each assay [31].

### 2.7. Determination of Oxidative Stress Markers

The levels of oxidative stress-related biomarkers in gastric tissues were evaluated by measuring superoxide dismutase (SOD) activity and reduced glutathione (GSH) content. Briefly, gastric tissues were rapidly excised, rinsed with ice-cold saline, and homogenized in phosphate-buffered saline (PBS, pH 7.4) under cold conditions. The homogenates were centrifuged at 12,000× *g* for 15 min at 4 °C, and the supernatants were collected for biochemical analysis. SOD and GSH assay kits were purchased from Cayman Chemical (Ann Arbor, MI, USA). The assay sensitivities were 0.5 U/mL for SOD and 0.04 μmol/L for GSH.The results were expressed as μmol per gram of tissue. Protein concentrations were determined using the Bradford method to normalize enzyme activity. All biochemical measurements were performed in triplicate technical replicates [32]. All assays were performed according to the manufacturers’ protocols. Data were expressed as mean ± standard deviation (*n* = 5 per group).

### 2.8. Western Blot Analysis

Western blot analysis was performed to evaluate the expression levels of inflammation-related proteins in gastric tissues. Briefly, gastric tissues were homogenized in RIPA lysis buffer containing protease and phosphatase inhibitors and centrifuged at 12,000× *g* for 15 min at 4 °C. The supernatants were collected, and protein concentrations were determined using the Bradford assay. Equal amounts of protein (20–30 μg) were separated by SDS–PAGE (Sodium Dodecyl Sulfate-polyacrylamide gel electrophoresis) and transferred onto polyvinylidene difluoride membranes. The membranes were blocked with 5% skim milk in Tris-buffered saline containing 0.1% Tween-20 for 1 h at room temperature (22–25 °C) and then incubated overnight at 4 °C with primary antibodies against inducible nitric oxide synthase (iNOS), cyclooxygenase-2 (COX-2), and β-actin. After washing, the membranes were incubated with appropriate horseradish peroxidase-conjugated secondary antibodies for 1 h at room temperature. Protein bands were visualized using enhanced chemiluminescence reagents and detected using an imaging system [33]. The band intensities were quantified using Image J software (version 1.53, National Institutes of Health, Bethesda, MD, USA) and normalized to β-actin as a loading control.

### 2.9. Histopathological Examination

On the last day of the experiment, animals were euthanized under isoflurane anesthesia, and the stomachs were excised for gross and histopathological evaluation. Gastric tissues were processed using standard histological procedures, including fixation, paraffin embedding, sectioning, and hematoxylin and eosin (H&E) staining. For each animal in the experimental group, the histopathological examination results were used to evaluate damage and atrophy of epithelial cells, ulcers, and inflammatory cell infiltration in the submucosal tissue. The histopathological lesion scores of the gastric mucosa were calculated according to the histopathological lesion scores index (Table 2) prepared by modifying the methods of Simões et al. and Zhao et al. [34,35]. The histopathological examination additionally evaluated characteristic acute gastric injury features, including mucosal erosion, epithelial damage, hemorrhage, and submucosal edema, which were qualitatively assessed in representative gastric tissue sections.

### 2.10. Gastric Gland Length

Gastric gland length was determined by observing gastric mucosal tissue using an Olympus DP70 microscope (Olympus Optical Co., Ltd., Tokyo, Japan) and measuring the length of gastric mucosal villi using an Image Measurement System [36]. The gastric gland length was measured for each prepared pathological tissue slide, and the mean score and standard deviation of each group were used as the villi length of the corresponding individual.

### 2.11. Immunohistochemistry Analysis

Gastric tissues were fixed in 10% neutral-buffered formalin, embedded in paraffin, and sectioned at a thickness of 4–5 μm. The sections were mounted on glass slides and dried prior to staining. The slides were then deparaffinized in xylene, rehydrated through a graded ethanol series, and subjected to antigen retrieval using 10 mM citrate buffer (pH 6.0). Endogenous peroxidase activity was blocked using 3% hydrogen peroxide. The sections were incubated overnight at 4 °C with anti-NF-κB p65 antibody (1:100 dilution; Cell Signaling Technology, Danvers, MA, USA), followed by a biotinylated secondary antibody and streptavidin–HRP. Color development was achieved using 3,3′-diaminobenzidine (DAB), and the slides were counterstained with hematoxylin. Stained sections were observed under a bright-field microscope (Olympus BX53, Olympus Optical Co., Ltd., Tokyo, Japan) [37].

### 2.12. Statistical Analysis

Data are presented as mean ± standard deviation (SD). In vitro experiments were performed independently at least three times. For in vivo experiments, each group consisted of five animals (*n* = 5). An a priori statistical power analysis was conducted based on previously published HCl/ethanol-induced gastric injury studies with similar experimental conditions and expected effect sizes. Although the present sample size (*n* = 5 per group) was sufficient to detect large treatment effects, it may have limited statistical power to identify moderate differences among treatment groups. Statistical analyses were performed using SPSS software version 12.0 (SPSS Inc., Chicago, IL, USA). Differences among groups were analyzed using one-way analysis of variance (ANOVA) followed by Duncan’s multiple range test for multiple comparisons. Statistical significance was accepted at *p* < 0.05.

## 3. Results

### 3.1. Evaluation of Clinical Symptoms

**Figure 1:**
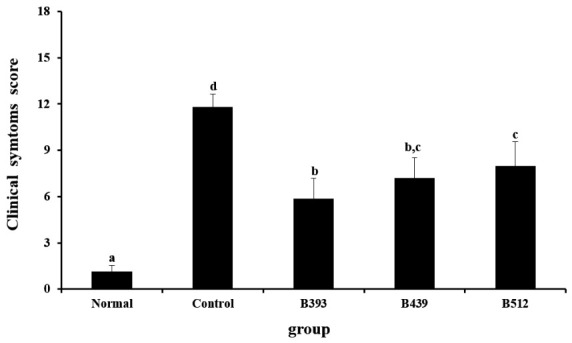
Effect of *Bacillus amyloliquefaciens* and *Bacillus subtilis* isolated from Cheonggukjang on clinical symptom scores. Normal, non-treated + DW; Control, 150 mM HCl/60% ethanol + DW; B393, 150 mM HCl/60% ethanol + *Bacillus amyloliquefaciens* C393 1.0 × 10^9^ CFU; B439, 150 mM HCl/60% ethanol + *Bacillus subtilis* C439 1.0 × 10^9^ CFU; B512, 150 mM HCl/60% ethanol + *Bacillus subtilis* C512 1.0 × 10^9^ CFU. Data are expressed as mean ± SD (*n* = 5). Different superscript letters indicate statistically significant differences among groups (*p* < 0.05) according to Duncan’s multiple range test following one-way ANOVA.

The results of clinical symptom evaluation are presented in Figure 1. The clinical symptom score was 1.16 ± 0.36 in the normal group, indicating normal behaviors, such as walking and grooming; however, in the control group administered HCl/ethanol (150 mM HCl + 60% ethanol) and distilled Water (DW), the score was 11.80 ± 0.84, indicating mostly no movement and only slight movement in response to stimulation. In contrast, compared to that in the control group, movement was significantly increased (*p* < 0.05) in the treatment groups: *B. amyloliquefaciens* C393 1.0 × 10^9^ CFU administration group (B393), *B. subtilis* C439 1.0 × 10^9^ CFU administration group (B439), and *B. subtilis* C512 1.0 × 10^9^ CFU administration, with scores of 5.88 ± 1.29, 7.20 ± 1.30, and 8.00 ± 1.58, respectively.

### 3.2. Measurement of Gastric Damage Inhibition Rate

**Figure 2:**
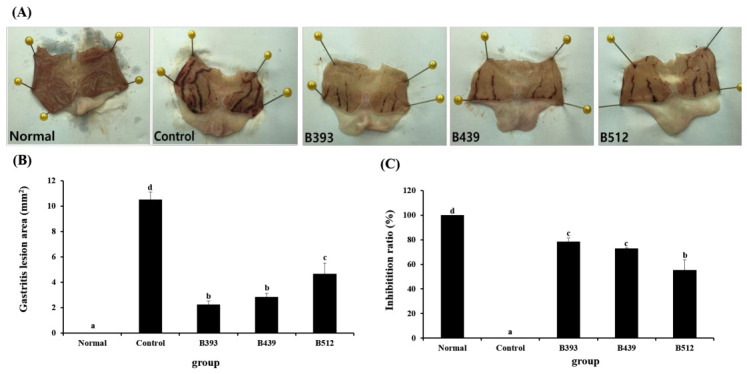
Protective effects of *Bacillus amyloliquefaciens* and *Bacillus subtilis* strains on HCl/ethanol-induced gastric mucosal injury in rats. (**A**) Gross lesion, (**B**) gastritis lesion area (mm^2^) and (**C**) inhibition ratio (%). Normal, non-treated + DW; Control, 150 mM HCl/60% ethanol + DW; B393, 150 mM HCl/60% ethanol + *Bacillus amyloliquefaciens* C393 1.0 × 10^9^ CFU; B439, 150 mM HCl/60% ethanol + *Bacillus subtilis* C439 1.0 × 10^9^ CFU; B512, 150 mM HCl/60% ethanol + *Bacillus subtilis* C512 1.0 × 10^9^ CFU. Data are expressed as mean ± SD (*n* = 5). Different superscript letters indicate statistically significant differences among groups (*p* < 0.05) according to Duncan’s multiple range test following one-way ANOVA.

Compared to that of the normal group, which was not administered HCl/ethanol, the control group, which was administered DW along with HCl/ethanol, showed a damage area of 10.52 ± 0.59, with distinct mucosal damage and band-shaped linear hemorrhages in the body and fundus of the stomach (Figure 2A). In contrast, the treatment groups B393, B439, and B512 showed damage areas of 2.25 ± 0.29, 2.85 ± 0.29, and 4.67 ± 0.83, respectively (Figure 2B), and inhibition rates of 78.6 ± 2.8%, 72.8 ± 0.5%, and 55.5 ± 7.9%, respectively (Figure 2C). Taken together, the groups administered *Bacillus* strains exhibited protective effects against HCl/ethanol-induced gastritis.

### 3.3. Effects on Gastric pH and Gastric Juice Volume

**Figure 3:**
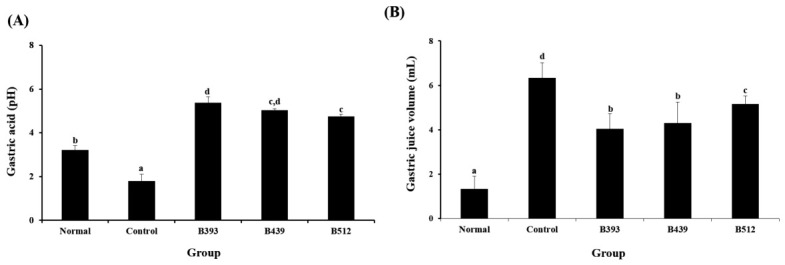
Effects of *Bacillus amyloliquefaciens* and *Bacillus subtilis* isolated from Cheonggukjang on (**A**) gastric juice pH and (**B**) gastric juice volume in rats with HCl/ethanol-induced gastric injury. Normal, non-treated + DW; Control, 150 mM HCl/60% ethanol + DW; B393, 150 mM HCl/60% ethanol + *Bacillus amyloliquefaciens* C393 1.0 × 10^9^ CFU; B439, 150 mM HCl/60% ethanol + *Bacillus subtilis* C439 1.0 × 10^9^ CFU; B512, 150 mM HCl/60% ethanol + *Bacillus subtilis* C512 1.0 × 10^9^ CFU. Data are expressed as mean ± SD (*n* = 5). Different superscript letters indicate statistically significant differences among groups (*p* < 0.05) according to Duncan’s multiple range test following one-way ANOVA.

The effects of *Bacillus* strains on gastric secretion parameters are presented in Figure 3. HCl/ethanol administration markedly altered gastric secretion, as evidenced by a significant increase in gastric juice volume and decrease in gastric pH compared with those of the normal group. The control group exhibited a gastric pH of 1.80 ± 0.30, indicating strong acidification. Pretreatment with *Bacillus* strains significantly restored gastric pH to normal levels. The B393 group showed the highest pH value (5.38 ± 0.27), followed by the B439 (5.04 ± 0.07) and B512 (4.75 ± 0.09) groups, indicating effective suppression of gastric acidification. Similarly, gastric juice volume was significantly elevated in the control group (6.33 ± 0.68 mL) compared with that in the normal group (1.33 ± 0.58 mL). Treatment with *Bacillus* strains markedly reduced gastric secretion volume. The B393 group exhibited the most pronounced reduction (4.03 ± 0.68 mL), followed by the B439 (4.30 ± 0.95 mL) group, while the B512 group showed a moderate decrease (5.17 ± 0.35 mL). These findings indicate that Cheonggukjang-derived *Bacillus* strains exert gastroprotective effects by regulating gastric acid secretion and reducing excessive gastric juice production, with B393 showing the strongest effect.

### 3.4. Effects on Serum Pro-Inflammatory Cytokines (TNF-α and IL-6)

**Figure 4:**
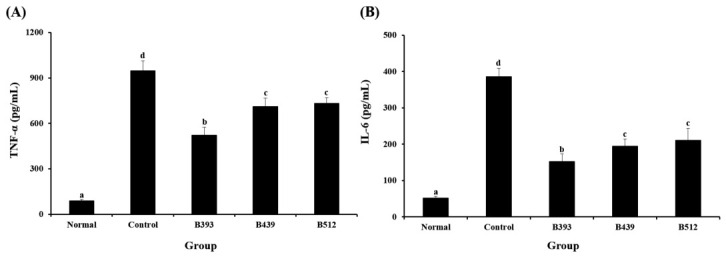
Effects of *Bacillus amyloliquefaciens* and *Bacillus subtilis* isolated from Cheonggukjang on serum (**A**) TNF-α and (**B**) IL-6 levels in rats with HCl/ethanol-induced gastric injury. Normal, non-treated + DW; Control, 150 mM HCl/60% ethanol + DW; B393, 150 mM HCl/60% ethanol + *Bacillus amyloliquefaciens* C393 1.0 × 10^9^ CFU; B439, 150 mM HCl/60% ethanol + *Bacillus subtilis* C439 1.0 × 10^9^ CFU; B512, 150 mM HCl/60% ethanol + *Bacillus subtilis* C512 1.0 × 10^9^ CFU. Data are expressed as mean ± SD (*n* = 5). Different superscript letters indicate statistically significant differences among groups (*p* < 0.05) according to Duncan’s multiple range test following one-way ANOVA.

The effects of Cheonggukjang-derived *Bacillus* strains on systemic inflammatory responses were evaluated by measuring serum levels of the pro-inflammatory cytokines TNF-α and IL-6 (Figure 4). HCl/ethanol administration significantly elevated serum TNF-α levels compared with those in the normal group. Specifically, TNF-α increased markedly from 87 ± 10 pg/mL in the normal group to 946 ± 67 pg/mL in the control group (*p* < 0.001), indicating a strong inflammatory response. Pretreatment with *Bacillus* strains significantly attenuated this increase. The B393 group showed the most pronounced reduction in TNF-α levels (521 ± 55 pg/mL, *p* < 0.001 vs. control), whereas the B439 and B512 groups exhibited moderate reductions (712 ± 55 and 733 ± 37 pg/mL, respectively; *p* < 0.01 vs. control). A similar trend was observed for IL-6 levels. The control group showed a significant increase (386 ± 23 pg/mL) compared with that in the normal group (52 ± 5 pg/mL, *p* < 0.001). *Bacillus* treatment significantly reduced IL-6 levels, with the B393 group demonstrating the strongest inhibitory effect (152 ± 21 pg/mL, *p* < 0.001 vs. control), followed by the B439 (195 ± 20 pg/mL) and B512 (211 ± 33 pg/mL) groups, both showing significant reductions (*p* < 0.01 vs. control). These results indicate that Cheonggukjang-derived *Bacillus* strains effectively suppress systemic inflammatory responses induced by HCl/ethanol, with B393 exhibiting the most potent anti-inflammatory activity among the tested strains.

### 3.5. Effects of Bacillus Strains on Oxidative Stress Markers (SOD and GSH)

**Figure 5:**
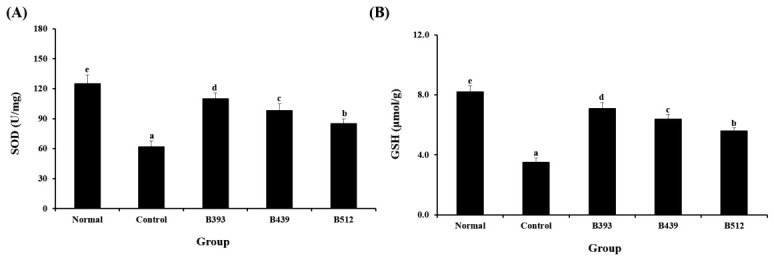
Effects of *Bacillus amyloliquefaciens* and *Bacillus subtilis* isolated from Cheonggukjang on (**A**) SOD activity and (**B**) GSH levels in rats with HCl/ethanol-induced gastric injury. Normal, non-treated + DW; Control, 150 mM HCl/60% ethanol + DW; B393, 150 mM HCl/60% ethanol + *Bacillus amyloliquefaciens* C393 1.0 × 10^9^ CFU; B439, 150 mM HCl/60% ethanol + *Bacillus subtilis* C439 1.0 × 10^9^ CFU, B512, 150 mM HCl/60% ethanol + *Bacillus subtilis* C512 1.0 × 10^9^ CFU. Data are expressed as mean ± SD (*n* = 5). Different superscript letters indicate statistically significant differences among groups (*p* < 0.05) according to Duncan’s multiple range test following one-way ANOVA.

The effects of Cheonggukjang-derived *Bacillus* strains on oxidative stress in gastric tissues were evaluated by measuring SOD activity and GSH levels (Figure 5). HCl/ethanol administration significantly impaired antioxidant defense systems, as evidenced by a marked decrease in SOD activity and GSH levels in the control group compared with those in the normal group. Specifically, SOD activity decreased from 125 ± 9 U/mg protein in the normal group to 62 ± 6 U/mg protein in the control group (*p* < 0.001). Pretreatment with *Bacillus* strains significantly restored SOD activity. The B393 group exhibited the highest recovery (110 ± 6 U/mg protein), followed by the B439 (98 ± 7 U/mg protein) and B512 (85 ± 5 U/mg protein) groups, indicating a strain-dependent antioxidant effect. Similarly, GSH levels were significantly reduced in the control group (3.5 ± 0.3 μmol/g tissue) compared with those in the normal group (8.2 ± 0.4 μmol/g tissue) (*p* < 0.001). *Bacillus* treatment effectively reversed this depletion. The B393 group showed the greatest restoration (7.1 ± 0.4 μmol/g tissue), followed by the B439 (6.4 ± 0.3 μmol/g tissue) and B512 (5.6 ± 0.2 μmol/g tissue) groups. These findings indicate that Cheonggukjang-derived *Bacillus* strains attenuate HCl/ethanol-induced oxidative stress by enhancing endogenous antioxidant defense systems, with B393 demonstrating the strongest protective effect.

### 3.6. Effects on Inflammatory Protein Expression

**Figure 6:**
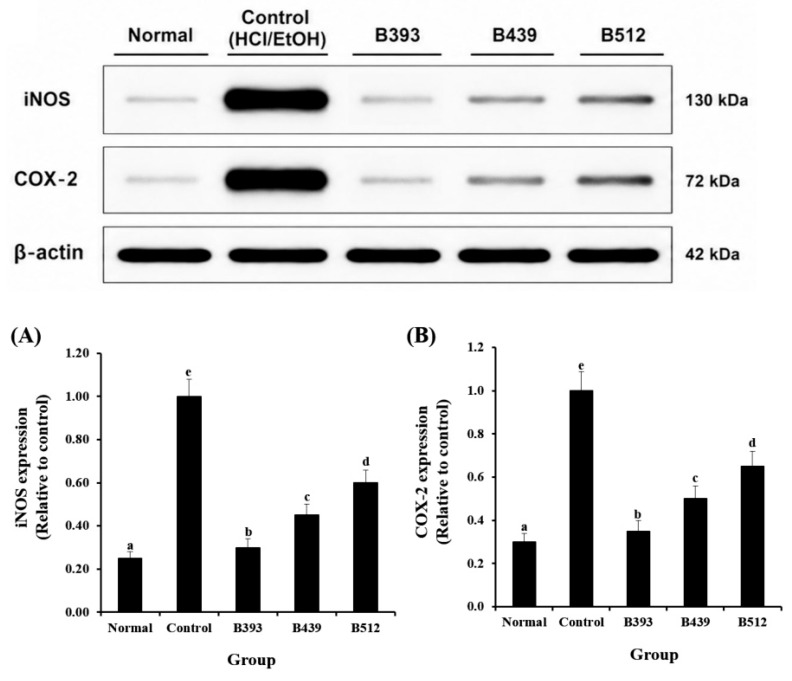
Cheonggukjang-derived *Bacillus* strains suppress HCl/ethanol-induced inflammatory protein expression. Densitometric analysis of (**A**) iNOS and (**B**) COX-2 protein expression normalized to β-actin. Normal, non-treated + DW; Control, 150 mM HCl/60% ethanol + DW; B393, 150 mM HCl/60% ethanol + *Bacillus amyloliquefaciens* C393 1.0 × 10^9^ CFU; B439, 150 mM HCl/60% ethanol + *Bacillus subtilis* C439 1.0 × 10^9^ CFU; B512, 150 mM HCl/60% ethanol + *Bacillus subtilis* C512 1.0 × 10^9^ CFU. Data are expressed as mean ± SD (*n* = 5). Different superscript letters indicate statistically significant differences among groups (*p* < 0.05) according to Duncan’s multiple range test following one-way ANOVA.

To investigate the anti-inflammatory effects of Cheonggukjang-derived *Bacillus* strains, the protein expression levels of iNOS and COX-2 were quantified by densitometric analysis of Western blot bands (Figure 6). Representative Western blot images from biological replicates (*n* = 5 per group) are shown. The expected molecular weights of the detected proteins were approximately 130 kDa for iNOS, 70 kDa for COX-2, and 42 kDa for β-actin. HCl/ethanol administration significantly increased the expression of inflammatory proteins compared with that in the normal group. Specifically, iNOS expression was markedly elevated in the control group (1.00 ± 0.08) compared with that in the normal group (0.25 ± 0.03, *p* < 0.001). Pretreatment with *Bacillus* strains significantly attenuated iNOS expression. Among the tested strains, the B393 group showed the greatest reduction in iNOS expression (0.30 ± 0.04, *p* < 0.001 vs. control), followed by the B439 group (0.45 ± 0.05, *p* < 0.001), whereas the B512 group exhibited a relatively weaker inhibitory effect (0.60 ± 0.06, *p* < 0.01). A similar trend was observed for COX-2 expression. The control group showed a significant increase (1.00 ± 0.09) compared with that in the normal group (0.30 ± 0.04, *p* < 0.001). *Bacillus* treatment significantly reduced COX-2 levels, with B393 showing the strongest inhibitory effect (0.35 ± 0.05, *p* < 0.001), followed by B439 (0.50 ± 0.06, *p* < 0.001) and B512 (0.65 ± 0.07, *p* < 0.01). These findings indicate that *Bacillus* strains suppress HCl/ethanol-induced inflammatory responses in a strain-dependent manner, with B393 exhibiting the most potent inhibitory effect on iNOS and COX-2 protein expression.

### 3.7. Histopathological Changes in Gastric Tissues

**Figure 7:**
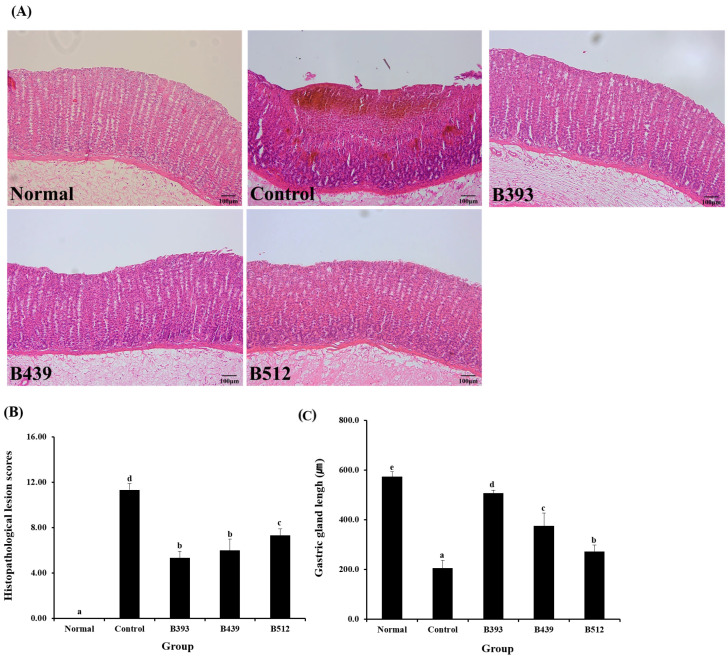
Histopathological analysis of gastric tissues following HCl/ethanol-induced injury. (**A**) Representative H&E-stained images of gastric tissues from each group. (**B**) Histopathological lesion scores. (**C**) Gastric gland length. Normal, non-treated + DW; Control, 150 mM HCl/60% ethanol + DW; B393, 150 mM HCl/60% ethanol + *Bacillus amyloliquefaciens* C393 1.0 × 10^9^ CFU; B439, 150 mM HCl/60% ethanol + *Bacillus subtilis* C439 1.0 × 10^9^ CFU; B512, 150 mM HCl/60% ethanol + *Bacillus subtilis* C512 1.0 × 10^9^ CFU. Data are expressed as mean ± SD (*n* = 5). Different superscript letters indicate statistically significant differences among groups (*p* < 0.05) according to Duncan’s multiple range test following one-way ANOVA.

Histopathological examination was conducted to assess the protective effects of *Bacillus* strains on HCl/ethanol-induced gastric mucosal injury (Figure 7). The normal group exhibited intact gastric mucosal architecture with well-organized epithelial layers and no signs of inflammation or hemorrhage. In contrast, the control group showed severe histopathological alterations, including extensive epithelial disruption, hemorrhage, mucosal erosion, and marked inflammatory cell infiltration, indicating significant gastric injury. Pretreatment with *Bacillus* strains markedly attenuated these histological abnormalities. The B393 and B439 groups showed substantial preservation of mucosal integrity, with reduced epithelial damage and inflammatory infiltration compared with those in the control group. Notably, the B439-treated group exhibited the most pronounced protective effect, displaying nearly normal mucosal architecture. The B512 group also demonstrated a protective effect; however, moderate epithelial disruption and inflammatory infiltration were still observed compared with those in the B393 and B439 groups. Quantitative histological scoring further confirmed these findings. The control group showed a significantly higher damage score than the normal group (*p* < 0.001), whereas all *Bacillus*-treated groups exhibited significantly reduced scores. Among them, the B439 group showed the lowest histological damage score, followed by the B393 and B512 groups.

### 3.8. Immunohistochemical Analysis of Inflammatory Markers

**Figure 8:**
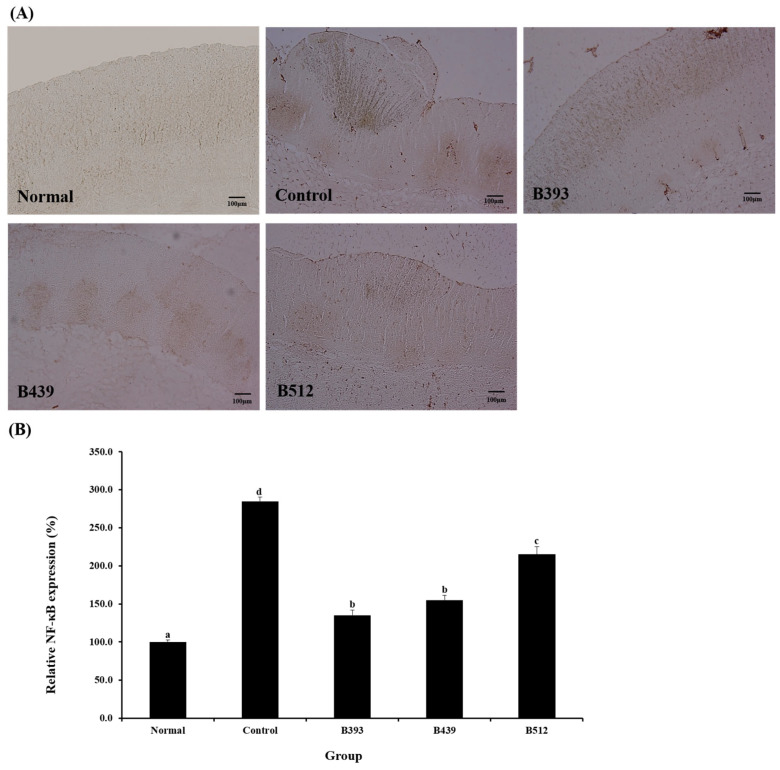
Immunohistochemical analysis of NF-κB p65 expression in gastric tissues following HCl/ethanol-induced gastric injury. (**A**) Representative immunohistochemical images of NF-κB p65 expression in gastric tissues. (**B**) Quantification of relative NF-κB p65 immunoreactivity using ImageJ software. Normal, non-treated + DW; Control, 150 mM HCl/60% ethanol + DW; B393, 150 mM HCl/60% ethanol + *Bacillus amyloliquefaciens* C393 1.0 × 10^9^ CFU; B439, 150 mM HCl/60% ethanol + *Bacillus subtilis* C439 1.0 × 10^9^ CFU; B512, 150 mM HCl-60% ethanol + *Bacillus subtilis* C512 1.0 × 10^9^ CFU. Data are expressed as mean ± SD (*n* = 5). Different superscript letters indicate statistically significant differences among groups (*p* < 0.05) according to Duncan’s multiple range test following one-way ANOVA.

Immunohistochemical analysis was performed to evaluate NF-κB p65 expression in gastric tissues following HCl/ethanol-induced gastric injury (Figure 8). Representative immunohistochemical staining images are shown in Figure 8A. In the normal group, only weak NF-κB p65 immunoreactivity was observed, indicating low basal expression in the gastric mucosa. In contrast, the control group exhibited markedly increased NF-κB p65-positive staining throughout the gastric mucosal layer, suggesting enhanced inflammatory responses following HCl/ethanol administration. Pretreatment with Cheonggukjang-derived *Bacillus* strains reduced NF-κB p65 immunoreactivity compared with that in the control group and partially restored the histological appearance of the gastric mucosa. The B393 and B439 groups showed marked reductions in staining intensity, whereas the B512 group also demonstrated decreased immunoreactivity compared with the control group. Among the treatment groups, the B439 group exhibited the lowest level of NF-κB p65-positive staining, approaching that observed in the normal group. To objectively evaluate NF-κB expression, quantitative image analysis was performed using Image J software on standardized images (Figure 8B). The percentage of NF-κB-positive staining area was significantly increased in the control group compared with that in the normal group (*p* < 0.001). In contrast, all *Bacillus*-treated groups exhibited significantly lower NF-κB-positive staining than the control group (*p* < 0.05). Among the treatment groups, B439 showed the greatest reduction in NF-κB immunoreactivity, followed by B393 and B512. These findings indicate that pretreatment with Cheonggukjang-derived *Bacillus* strains attenuated NF-κB p65 expression in gastric tissues following HCl/ethanol-induced gastric injury and may contribute to the reduction of inflammatory responses in the gastric mucosa.

## 4. Discussion

HCl/ethanol administration significantly increased gastric lesion severity, decreased gastric pH, and elevated gastric secretion volume, indicating disruption of gastric homeostasis. These findings are consistent with previous reports showing that excessive gastric acid and mucosal erosion are key contributors to acute gastric injury. This pathological condition is primarily associated with an imbalance between aggressive factors, such as gastric acid and ROS, and mucosal defense mechanisms [38].

Oxidative stress plays a central role in gastric mucosal injury through excessive ROS generation and lipid peroxidation [39]. Microbial-derived bioactive compounds reportedly enhance antioxidant defense systems and exert cytoprotective effects [40]. Herein, the marked decrease in antioxidant markers, including SOD and GSH, observed in the control group confirms the involvement of oxidative stress in this model. The restoration of these antioxidant defenses by *Bacillus* strains suggests their protective role in scavenging ROS and maintaining redox balance. Notably, the B393 strain exhibited the most potent antioxidant effect, suggesting strain-specific functional differences. The assessment of oxidative stress was limited to antioxidant defense markers (SOD and GSH). Direct evaluation of lipid peroxidation markers, including malondialdehyde (MDA) and reactive oxygen species (ROS), was not performed and should be included in future studies to provide a more comprehensive characterization of oxidative injury.

Pro-inflammatory cytokines, including TNF-α and IL-6, are key mediators of gastric inflammation and contribute to mucosal damage [41]. The elevated levels of these cytokines in the control group are consistent with the activation of systemic inflammatory responses during gastric injury. Further, the significant reduction of TNF-α and IL-6 levels following *Bacillus* treatment indicates effective suppression of inflammatory signaling. Among the tested strains, B393 exhibited the strongest effects on gastric pH restoration, reduction of gastric juice volume, recovery of antioxidant markers, and suppression of serum TNF-α and IL-6 levels. These findings suggest that B393 may exert its gastroprotective activity mainly through enhancement of antioxidant defense and attenuation of systemic inflammatory responses. Also, B393 showed the most pronounced reduction in iNOS and COX-2 protein expression and provided superior histological protection, as evidenced by better preservation of gastric mucosal architecture and reduced inflammatory infiltration. This indicates that B393 may have a stronger influence on local inflammatory mediator regulation and tissue-level mucosal recovery. B512 also showed gastroprotective effects, but its efficacy was generally weaker than that of B393 and B439. Therefore, the present findings suggest that Cheonggukjang-derived *Bacillus* strains exert strain-specific and partially complementary protective effects against HCl/ethanol-induced gastric injury. Consistent with the present findings, previous studies have demonstrated that attenuation of inflammatory cytokines contributes significantly to gastroprotection. For example, naringenin reduced ethanol-induced gastric ulceration by decreasing TNF-α, IL-6, NF-κB activation, myeloperoxidase activity, and oxidative stress markers [42]. Similarly, sinapic acid ameliorated ethanol-induced gastric mucosal injury through suppression of pro-inflammatory cytokines and oxidative stress responses [43]. Oxyresveratrol has also been reported to improve gastric mucosal integrity by reducing TNF-α, IL-6, NF-κB, and COX-2 expression levels in experimental gastric injury models [44]. These findings support the interpretation that the cytokine-lowering effects observed in the B393 group may contribute to its gastroprotective activity.

**Figure 9:**
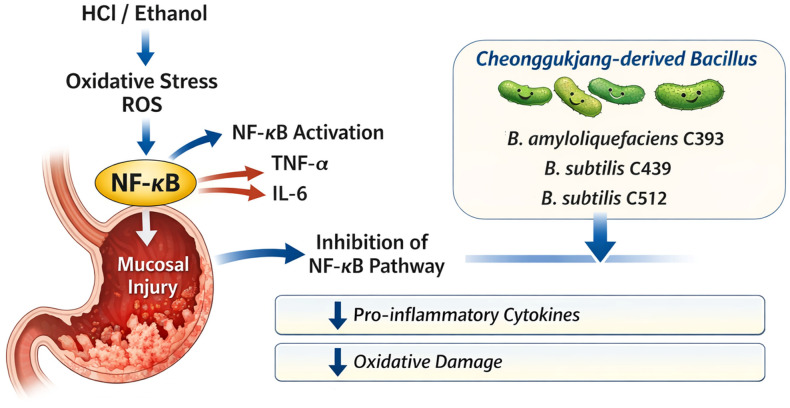
Proposed mechanism underlying the gastroprotective effects of Cheonggukjang-derived *Bacillus* strains against HCl/ethanol-induced gastric mucosal injury.

Inducible inflammatory enzymes, such as iNOS and COX-2, are regulated by the NF-κB signaling pathway and are critically involved in gastric injury [45]. Overexpression of these proteins contributes to excessive nitric oxide and prostaglandin production, leading to tissue damage. Herein, Western blot analysis revealed that HCl/ethanol administration significantly increased iNOS and COX-2 expression, whereas *Bacillus* treatment markedly suppressed their expression. NF-κB is a central transcription factor controlling inflammatory responses and is a major therapeutic target in gastric diseases [46]. The differential efficacy profiles of B393 and B439 may reflect strain-specific biological properties. B393 showed stronger restoration of SOD and GSH and greater suppression of serum TNF-α and IL-6, suggesting more effective regulation of oxidative stress and systemic inflammation. In contrast, B439 showed stronger suppression of iNOS and COX-2 expression and greater histological preservation, suggesting a stronger influence on local inflammatory responses and tissue-level protection. However, because bacterial metabolite profiling and direct signaling pathway analyses were not performed, the mechanisms responsible for these strain-specific differences remain to be elucidated. Its activation leads to the amplification of inflammatory cascades and subsequent tissue injury. Mechanistically, the inhibition of these inflammatory mediators observed herein suggests that *Bacillus* strains exert their protective effects by regulating NF-κB-dependent signaling pathways (Figure 9). This was further supported by immunohistochemical analysis. Specifically, the analysis demonstrated that NF-κB activation was significantly elevated in the control group and effectively suppressed in the *Bacillus*-treated groups. Moreover, it revealed severe mucosal damage, hemorrhage, and inflammatory infiltration in the control group, whereas *Bacillus*-treated groups exhibited significant preservation of mucosal integrity. Gastric mucus secretion and prostaglandin E_2_ (PGE_2_) are major protective factors that maintain gastric mucosal integrity by regulating bicarbonate secretion, mucosal blood flow, and epithelial restitution following injury [47]. In addition, epithelial barrier function is critically regulated by tight junction proteins, including occludin, claudins, and zonula occludens-1 (ZO-1), and disruption of these proteins has been associated with increased mucosal permeability and tissue damage [48]. Previous studies have also demonstrated that probiotics can enhance epithelial barrier integrity through modulation of tight junction-associated pathways and mucosal defense mechanisms [49]. Therefore, the absence of measurements related to gastric mucus production, PGE_2_ levels, and tight junction protein expression represents a limitation of the present study. Future studies incorporating these parameters will be necessary to further clarify whether the observed gastroprotective effects are mediated through enhancement of gastric mucosal barrier function. The objective of the present study was not pharmacological validation of gastroprotection, but comparative evaluation of strain-specific probiotic activities. Therefore, the absence of a drug control does not affect the interpretation of the relative efficacy among the tested *Bacillus* strains, although it limits comparison with clinically used gastroprotective agents. Only male Sprague–Dawley rats were included in this study. Although this approach reduced biological variability associated with the estrous cycle, sex-dependent differences in gastric injury susceptibility, inflammatory responses, and probiotic efficacy cannot be excluded. Future studies incorporating both male and female animals are warranted to improve the generalizability of the findings. Also, a limitation of the present study is the relatively small sample size (*n* = 5 per group), which may reduce the statistical power for detecting moderate biological effects. Therefore, the current findings should be interpreted as preliminary evidence of gastroprotective activity. Further studies using larger experimental cohorts are necessary to confirm the reproducibility and translational applicability of these results.

Although reduced NF-κB p65 immunoreactivity in gastric tissues and decreased expression of NF-κB-regulated inflammatory mediators were observed following treatment with Cheonggukjang-derived *Bacillus* strains, direct assessment of NF-κB pathway activation was not conducted in the present study. Specifically, key signaling molecules involved in canonical NF-κB activation, including phosphorylated NF-κB p65 (p-p65), IκBα, and phosphorylated IκBα (p-IκBα), were not evaluated. Therefore, the present findings should be interpreted as indirect evidence suggesting a possible association between *Bacillus* treatment and modulation of NF-κB-related inflammatory responses rather than definitive proof of NF-κB pathway inhibition. Further mechanistic studies incorporating Western blot or molecular signaling analyses are required to confirm the precise involvement of NF-κB signaling in the observed gastroprotective effects. Previous studies have reported gastroprotective effects of *Bacillus* probiotics, while the present study provides comparative evidence regarding strain-specific differences among Cheonggukjang-derived *Bacillus* isolates under identical experimental conditions. Among the tested strains, B393 consistently showed the strongest antioxidant and anti-inflammatory effects, whereas B439 exhibited superior histological protection, suggesting that different strains may act through distinct but complementary mechanisms. The primary objective of the present study was to compare the strain-specific gastroprotective and anti-inflammatory activities of Cheonggukjang-derived *Bacillus* isolates rather than to establish therapeutic equivalence to existing anti-ulcer medications. The observed differences among B393, B439, and B512 indicate that probiotic-mediated gastroprotection is highly strain-dependent and may involve distinct biological mechanisms. A clinically established gastroprotective agent such as omeprazole or rebamipide was not included as a positive control. Because the primary purpose of this study was to compare strain-specific biological activities among Cheonggukjang-derived *Bacillus* isolates, direct comparison with conventional anti-ulcer drugs was not performed. However, this does not affect the interpretation of the relative efficacy among the tested *Bacillus* strains. Nevertheless, the absence of a pharmacological positive control may limit direct comparison with clinically established gastroprotective therapies. Future studies should be conducted to include a pharmacological reference group, such as omeprazole or rebamipide, to further strengthen the translational relevance of the present findings. Although the present findings demonstrate significant gastroprotective effects of Cheonggukjang-derived *Bacillus* strains against HCl/ethanol-induced gastric injury, the results should be interpreted with caution. The present study focused primarily on efficacy-related endpoints and did not include dedicated acute, subchronic, or chronic toxicity evaluations. Although many *Bacillus* species have been widely used as probiotics and are generally considered safe, safety characteristics are strain-dependent and require individual assessment before clinical application [50,51]. Therefore, the current findings support the potential of these strains as candidate gastroprotective probiotics rather than therapeutic agents. Further studies involving comprehensive toxicological assessments, long-term safety evaluations, and dose-escalation studies are warranted before consideration of clinical translation. In light of these findings, several limitations should be acknowledged. First, the sample size was relatively small, which may limit statistical power. Second, only male rats were used; therefore, sex-dependent differences could not be evaluated. Third, only a single dose of each *Bacillus* strain was tested, and dose–response relationships were not determined. Fourth, a standard gastroprotective drug was not included as a positive control. Finally, although NF-κB-related inflammatory responses were evaluated, direct mechanistic validation of the NF-κB signaling pathway was not performed.

Therefore, the present findings should be regarded as comparative preclinical evidence supporting the selection of promising probiotic candidates for future mechanistic and translational studies.

## 5. Conclusions

The collective evidence from this study demonstrates that Cheonggukjang-derived *Bacillus* strains exhibited protective effects against HCl/ethanol-induced gastric mucosal injury. B393 showed stronger antioxidant and cytokine-suppressive activities, whereas B439 demonstrated greater inhibition of inflammatory protein expression and superior histological protection. These findings suggest that Cheonggukjang-derived *Bacillus* strains may attenuate gastric injury through strain-specific antioxidant and anti-inflammatory activities.

## Figures and Tables

**Table 1:** Experimental design of the animal study.

Group	Material	Dose	*n*
Normal	Non-treated	-	5
Control	HCI/ethanol + Distilled Water	-	5
B393	HCI/ethanol + *Bacillus amyloliquefaciens* C393	1.0 × 10^9^ CFU	5
B439	HCI/ethanol + *Bacillus subtilis* C439	1.0 × 10^9^ CFU	5
B512	HCI/ethanol + *Bacillus subtilis* C512	1.0 × 10^9^ CFU	5

**Table 2:** Grading criteria for histopathological lesion scores of the stomach in SD rats.

Parameter	Score	Criteria
Inflammation	0	No lymphocytic or granulocytic infiltration
	1	Mild mucosal lymphocytic infiltration
	2	Moderate mucosal lymphocytic infiltration, some multifocal mucosal lymphoid aggregates
	3	Extensive multifocal mucosal lymphoid aggregates
	4	Multifocal mucosal and submucosal lymphoid aggregates
Atrophic gastritis	0	Parietal cell and glandular architecture preserved
	1	Minimal parietal cell loss, glandular architecture preserved
	2	Moderate parietal cell loss, glandular architecture preserved
	3	Significant parietal cell loss, glandular branching and hyperplasia
	4	Significant parietal cell loss, glandular branching and hyperplasia with submucosal glandular herniation

## Data Availability

The data presented in this study are available upon reasonable request from the corresponding author.
